# The MDM2 309T>G Polymorphism and Ovarian Cancer Risk: A Meta-Analysis of 1534 Cases and 2211 Controls

**DOI:** 10.1371/journal.pone.0055019

**Published:** 2013-01-31

**Authors:** Ying-Yu Ma, Tian-Pei Guan, Hai-Bo Yao, Sheng Yu, Le-Gao Chen, Ying-Jie Xia, Xu-Jun He, Hui-Ju Wang, Xiao-Ting Jiang, Hou-Quan Tao

**Affiliations:** 1 Key Laboratory of Gastroenterology of Zhejiang Province, Zhejiang Provincial People’s Hospital, Hangzhou, China; 2 Department of Surgical, Wenzhou Medical College, Wenzhou, China; 3 Clinical Laboratory, Zhejiang Provincial People’s Hospital, Hangzhou, China; 4 Department of Gastrointestinal Surgery, Zhejiang Provincial People’s Hospital, Hangzhou, China; IPO, Inst Port Oncology, Portugal

## Abstract

**Background:**

Recently, there have been a number of studies on the association between MDM2 (Murine Double Minute 2) 309 polymorphism and ovarian cancer risk. However, the results of previous reports remain controversial and ambiguous. Thus, we performed a meta-analysis to explore more precisely the association between MDM2 309 polymorphism and the risk of ovarian cancer.

**Methods:**

A meta-analysis was performed to examine the association between MDM2 309T>G polymorphism and ovarian cancer risk. Odds ratio (OR) and its 95% confidence interval (CI) were used for statistical analysis.

**Results:**

Our publication search identified a total of 6 studies with 1534 cases and 2211 controls. No significant association was found between MDM2 309T>G polymorphism and ovarian cancer risk in total population analysis. In the subgroup meta-analysis by ethnicity, a negative association was shown in Asian subgroup (G vs. T OR = 0.774, 95% CI = 0.628–0.955, *P* = 0.017, *P*_het_ = 0.327; GG vs. TT: OR = 0.601, 95% CI = 0.395–0.914, P = 0.017, *P*_het_ = 0.417; dominant model TG+GG vs. TT: OR = 0.661, 95% CI = 0.468–0.934, *P* = 0.019, *P*_het_ = 0.880), and no significant association in any genetic models among Caucasians was observed.

**Conclusions:**

This meta-analysis provides evidence for the association between MDM2 309 polymorphism and ovarian cancer risk, supporting the hypothesis that MDM2 SNP309 G allele acts as an important ovarian cancer protective factor in Asians but not in Caucasians.

## Introduction

Epithelial ovarian cancer (OC) is the leading cause of death from gynecologic malignancies. OC is mostly asymptomatic at early-stage, and most of the cases are diagnosed when the tumor has established regional or distant metastases [1].

Therefore, it is important to clarify the molecular mechanism of OC development which can help to detect OC at an early stage, and studies on gene polymorphism that affects the pathways known to influence the neoplastic process may be particularly relevant.

P53 is a tumor suppressor gene, which is involved in multiple pathways including apoptosis, cellular transcriptional control, and cell cycle regulation [2], [3]. MDM2 (mouse double minute 2 homolog) is a crucial negative regulator of the tumor suppressor p53. P53 and MDM2 act in a feedback loop where p53 activates MDM2 at the transcriptional level while MDM2 binds to the N -terminus of p53 protein, inhibits its activity and mediates its location and degradation through E3 ligase activity [4], [5], [6]. The expression level of MDM2 can be affected by several factors, one of which is single nucleotide polymorphism.

In 2004, Bond et al. reported that a polymorphism (SNP309T>G; rs2279744) in the MDM2 intronic promoter P2 affects MDM2 expression levels [7]. SNP309 (rs2279744) enhances the DNA-binding affinity of the transcriptional activator Sp1. This results in elevated MDM2 levels and consequently an attenuation of the p53 pathway associated with susceptibility to certain types of cancer [7], [8], [9], [10], [11], [12]. Following the discovery of the 309 polymorphism, conflicting evidence has linked the G-allele to enhanced cancer risk as well as early cancer diagnosis across different tumor types and ethnic groups [13], [14].

Other meta-analyses suggest that the G/G genotype is associated with an increased risk for lung, endometrial, and hepatocellular carcinomas, but not for breast or colorectal carcinomas [15], [16], [17]. Studies have also found that SNP309G is associated with early diagnosis of several malignancies in females but not in males [7], [18], [19]. Over the last two decades, a number of case–control studies were conducted to investigate the association between MDM2 polymorphism and ovarian cancer risk in humans. However,no quantitative summary of evidence has ever been developed so far since these studies reported conflicting results. The purpose of this meta-analysis is to provide a quantitative summary of evidence.

## Materials and Methods

### Publication Search

Computer searches were performed independently by two authors, covering all papers published in PubMed, Embase, Medline and Google Scholar before May 2012. The keywords were as follows: ovarian cancer/ovarian carcinoma, polymorphism/variant/genotype/SNP, and murine double minute 2/MDM2. The reference lists of the retrieved articles were hand-searched to obtain other relevant publications. All associated publications were evaluated to identify the most eligible literature. The results were limited to papers published in English.

### Inclusion and Exclusion Criteria

The following criteria were used to select studies for further meta-analysis: (1) the studies were case-control study; (2) the studies were about MDM2 309T>G polymorphism and risk of ovarian cancer; (3) the studies contained at least two comparison groups (cancer group vs. control group); (4) the studies included detailed genotyping data.

### Data Extraction

The extraction of data from all eligible publications was performed by two investigators independently, according to the inclusion and exclusion criteria listed above. For each study, information extracted were author’s last name, year of publication, country of origin, ethnicity, cancer type, sources of control and case groups, specimen of cases, genotyping methods for MDM2 SNP309T/G, total number of cases and controls as well as number of cases and controls with T/T, T/G and G/G genotypes. All the case and control groups were well controlled. The non-cancer controls had no history of gynecologic disease, and there was no present evidence of gynecologic cancer, any malignant disease or genetic disease. All case patients and control subjects were unrelated. There was no statistically significant difference in terms of age distribution, smoking habits or menstrual status between case and control groups.

### Statistical Analysis

Hardy-Weinberg equilibrium (HWE) for the control group of each study was assessed using goodness-of-fit test (χ2 of Fisher’s exact test). Based on both fixed effects and random-effects models, a pooled OR with 95% CI was used to assess the strength of association between MDM2 SNP309T/G polymorphism and ovarian cancer risk, depending on the heterogeneity of the analysis. In the overall and the subgroup meta-analysis, pooled ORs and 95% CIs for GG vs. TT, TG vs. TT, dominant model (TG+GG vs. TT), and recessive model (GG vs. TG+TT) were all calculated. Heterogeneity was assessed using Q-test and *I^2^* score. If the result of heterogeneity test was *P*>0.1, ORs were pooled according to the fixed-effects model (Mantel-Haenszel model). Otherwise, ORs were pooled according to the random-effects model (DerSimonian and Laird model). I^2^ was used to qualify variation in OR attributable to heterogeneity.

Publication bias was assessed using Egger test and Begg test. All statistical tests were performed using the software STATA v.10.0 (Stata Corporation, College Station, TX, USA). The results were considered statistically significant if *P*<0.05.

## Results

### Study Selection

**Figure 1:**
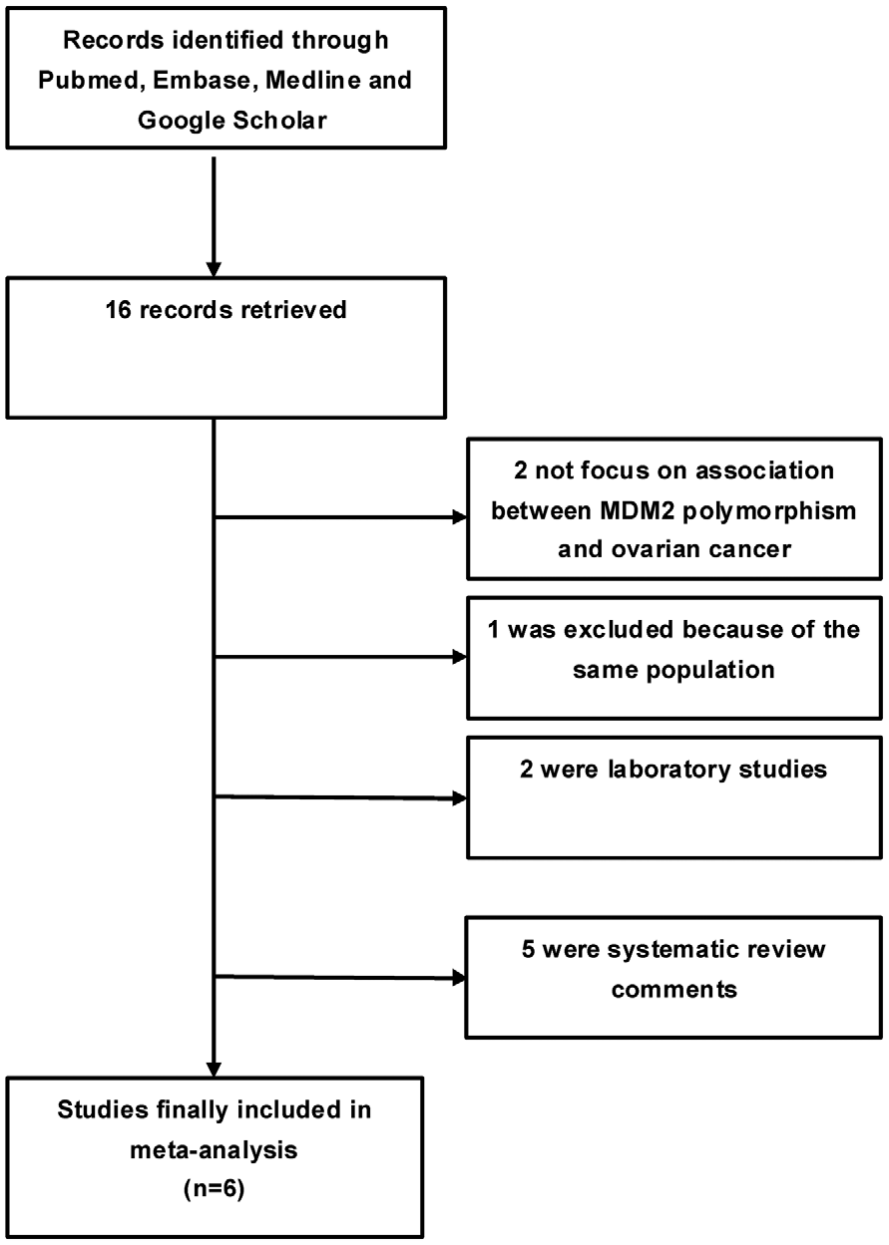
Flow chart of study study selection based on the inclusion and exclusion criteria.

A total of 16 records that fulfilled our search criteria were preliminarily identified for further detailed evaluation, which excluded ten studies (Figure 1). Two studies [20], [21] were not focused on MDM2 SNP309T/G polymorphism and ovarian cancer risk. One study (a conference abstract) [22] was excluded because it used the same population as an included study [23]. Two others [24], [25] were laboratory studies, and the rest of the 5 studies [17], [26], [27], [28], [29] were systematic review comments. At last, six studies [23], [30], [31], [32], [33], [34] on MDM2 SNP309 genotypes and ovarian cancer risk were identified, including a total of 1534 ovarian cancer cases and 2211 controls.

### Study Characteristics

Characteristics of the studies included in this meta-analysis are presented in Table I. All studies are case-control studies. Of these 6 studies, 2 used allele specific PCR, 2 used PCR-RFLP and 2 used pyrosequencing. The studies were carried out in Japan, UK, China, Norway and Czech Republic. Two studies were on Asians and four studies were on Caucasians. The studies carried out in China and Japan were used in Asian subgroup, and others were used in Caucasian subgroup. The distribution of genotypes in the controls was consistent with Hardy-Weinberg equilibrium (*P*>0.05) in all studies except for one study of Ueda et al. (*P* = 0.021) [34].

**Table 1:** MDM2 SNP309T>G Genotype Distribution and Allele Frequency in Cases and Controls.

Author-Year	Country	Genotype (N)								Allele frequency (N, %)				*P* HWE Controls
		Case				Control				Case		Control		
		total	TT	TG	GG	total	TT	TG	GG	T	G	T	G	
Kang et al. (2006)	China	257	77	120	60	257	56	121	80	274(53.3%)	240(46.7%)	233(45.3%)	281(54.7%)	0.422
Knappskog et al. (2011)	Norway	832	296	437	99	1337	561	617	159	1029(61.8%)	635(38.2%)	1739(65.0%)	935(34.9%)	0.591
Ueda et al. (2009)	Japan	85	21	45	19	108	20	66	22	87(51.2%)	83(48.8%)	106(49.1%)	110(50.9%)	0.021
Campbell et al. (2006)	UK	302	117	133	52	258	105	111	42	367(60.8%)	237(39.2%)	321(62.2%)	195(37.8%)	0.172
Copson et al. (2006)	UK	14	6	4	4	102	48	38	16	16(57.1%)	12(42.9%)	134(65.7%)	70(34.3%)	0.079
Krekac et al. (2008)	Czech Republic	44	24	18	2	149	61	71	17	66(75.0%)	22(25.0%)	193(64.8%)	105(35.2%)	0.591

### Quantitative Data Synthesis

**Figure 2:**
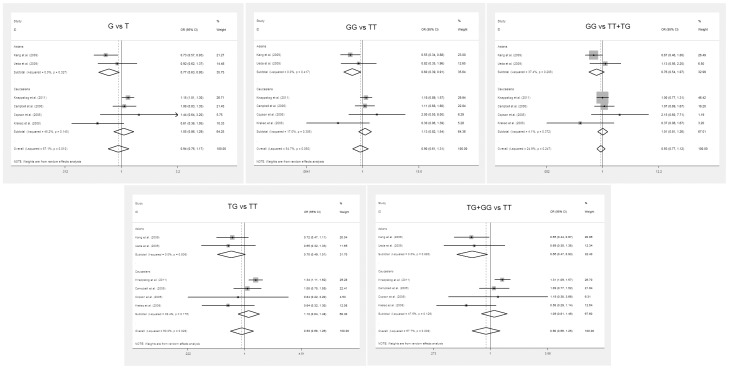
Forest plots of MDM2 309T/G polymorphism in ovarian cancer vs. normal control and subgroup analyses. The squares and horizontal lines correspond to the study specific OR and 95% CI. The area of the squares reflects the weight (inverse of the variance). The diamond represents the summary OR and 95% CI. OR: odds ratio.

The results on the associations between MDM2 SNP309 polymorphism and ovarian cancer risk, and of the heterogeneity test are shown in Table 2. The combined results based on all studies showed that variant genotypes are not associated with increased ovarian cancer risk in different genetic models (OR = 0.942, 95% CI = 0.760–1.167 for G vs. T, *P* = 0.583; OR = 0.895, 95% CI = 0.611–1.313 for GG vs. TT, *P* = 0.571; OR = 0.929, 95% CI = 0.684–1.261 for TG vs. TT, *P* = 0.635; OR = 0.905, 95% CI = 0.657–1.246 for the dominant model TG+GG vs. TT, *P* = 0.540; OR = 0.927, 95% CI = 0.770–1.116 for the recessive model GG vs. TT+TG, *P* = 0.424) (Figure 2). In the subgroup analysis by ethnicity, in Asian population, the results revealed significant associations between the MDM2 SNP309 polymorphism and ovarian cancer in three genotype distributions (G vs. T: OR = 0.774, 95% CI = 0.628–0.955, *P* = 0.017, *P*_het_ = 0.327; GG vs. TT: OR = 0.601, 95% CI = 0.395–0.914, *P* = 0.017, *P*_het_ = 0.417; dominant model TG+GG vs. TT: OR = 0.661, 95% CI = 0.468–0.934, *P* = 0.019, *P*_het_ = 0.880), but not in the other two genotype distributions (TG vs. TT: OR = 0.702, 95%CI = 0.486–1.013, *P* = 0.059, *P*_het_ = 0.806; GG vs. TT+TG: OR = 0.763, 95%CI = 0.543–1.072, *P* = 0.119, *P*_het_ = 0.206). In contrast, no significant association in any genetic models was observed in Caucasians (G vs. T: OR = 1.053, 95% CI = 0.856–1.294, *P* = 0.626, *P*_het_ = 0.140; GG vs. TT: OR = 1.125, 95% CI = 0.823–1.538, *P* = 0.459, *P*_het_ = 0.306; dominant model TG+GG vs. TT: OR = 1.091, 95% CI = 0.814–1.462, *P* = 0.560, *P*_het_ = 0.126; TG vs. TT: OR = 1.115, 95%CI = 0.840–1.480, *P* = 0.450, *P*_het_ = 0.176; GG vs. TT+TG: OR = 1.008, 95%CI = 0.807–1.258, *P* = 0.946, *P*_het_ = 0.372).

**Table 2:** Meta-analysis of the association between MDM2 SNP309 polymorphism and ovarian cancer risk.

Comparisons	Odds ratio	95%Confidence Interval	*P* value	Heterogeneity		Effects model
				I^2^	*P* value	
G vs T	0.942	0.760–1.167	0.583	67.1%	0.010	Random
Asians	0.774	0.628–0.955	0.017	0.0%	0.327	
Caucasians	1.053	0.856–1.294	0.626	45.2%	0.140	
GG vs TT	0.895	0.611–1.313	0.571	54.7%	0.050	Random
Asians	0.601	0.395–0.914	0.017	0.0%	0.417	
Caucasians	1.125	0.823–1.538	0.459	17.0%	0.306	
TG vs TT	0.929	0.684–1.261	0.635	60.0%	0.028	Random
Asians	0.702	0.486–1.013	0.059	0.0%	0.806	
Caucasians	1.115	0.840–1.480	0.450	39.4%	0.176	
TG+GG vs TT	0.905	0.657–1.246	0.540	67.7%	0.008	Random
Asians	0.661	0.468–0.934	0.019	0.0%	0.880	
Caucasians	1.091	0.814–1.462	0.560	47.5%	0.126	
GG vs TT+TG	0.927	0.770–1.116	0.424	24.9%	0.247	Fixed
Asians	0.763	0.543–1.072	0.119	37.4%	0.206	
Caucasians	1.008	0.807–1.258	0.946	4.1%	0.372	

### Tests of Heterogeneity

Statistically significant heterogeneity was observed between trials of the following analyses using Q statistic (G vs. T: *P* = 0.010, I^2^ = 67.1%; GG vs. TT: *P* = 0.050, I^2^ = 54.7%; TG vs. TT: *P* = 0.028, I^2^ = 60.0%; dominant model TG+GG vs. TT: *P* = 0.008, I^2^ = 67.7%) (Table 2), and the random-effects model was employed in these studies. We did not find the significant heterogeneity for the recessive model GG vs. TT+TG (*P* = 0.247, I^2^ = 24.9%), and a fixed-effects model was performed.

### Publication Bias

Begg’s test and Egger’s test and were performed to assess publication bias. Egger weighted regression method did not indicate evidence for publication bias for four of the five genetic models (G vs. T: *P* = 0.354; GG vs. TT: *P* = 0.679; dominant model TG+GG vs. TT: *P* = 0.063; recessive model GG vs. TT+TG: *P* = 0.656). This result was confirmed by Begg rank correlation method (G vs. T: *P* = 0.707; GG vs. TT: *P* = 0.707; TG vs. TT: *P* = 0.707; dominant model TG+GG vs. TT: *P* = 0.707; recessive model GG vs. TT+TG: *P* = 0.707) (Table 3).

**Table 3:** Publication bias test for MDM2 SNP309 polymorphism.

Comparisons	Egger test			Begg test*P* value
	Coefficient	*P* value	95% CI	
G vs T	−1.427	0.354	−5.209–2.356	0.707
GG vs TT	−0.644	0.679	–4.659–3.370	0.707
TG vs TT	−2.764	0.014	−4.587–0.941	0.707
TG+GG vs TT	−2.574	0.063	−5.367–0.219	0.707
GG vs TT+TG	0.641	0.656	−3.063–4.345	0.707

## Discussion

It has been discovered that a variant of SNP309G affects Sp1 binding to the MDM2 P2 promoter, which results in increased MDM2 transcript and protein [7]. After this discovery, studies described that the 309G status is associated with an early diagnosis and tumor formation in Li-Fraumeni syndrome and several malignancies, including breast cancer, soft tissue sarcomas, large cell lymphomas and colorectal cancer [7], [18], [19]. Interestingly, these associations were observed among females only, showing that SNP309G status could be due to the effects of gender-specific hormones. Another study further supported the fact that SNP309G’s impact on age of cancer onset is the largest among women below the average age of menopause [18], [19]. As ovarian cancer is largely hormone related, it is important to study the impact of MDM2 309 polymorphism on women with ovarian cancer.

Previous investigations found that the frequency distribution of SNP309G allele is significantly varied among different ethnicities, which led to conflicting evidence on the association between MDM2 309 T/G polymorphism and risk of cancer, particularly in Caucasian populations [13], [14]. It was also found that the G/G genotype is associated with an increased risk for lung, endometrial, and hepatocellular carcinomas, but not for breast or colorectal carcinomas [15], [16], [17]. Another study also showed that MDM2 SNP309 G allele probably acts as an important head and neck squamous cell carcinoma (HNSCC) protective factor in Caucasians, but not in Asians [35]. As conflicting results between studies or ethnic groups have been reported, it is necessary to make a quantitative summary to evaluate MDM2 309 T/G polymorphism and risk of cancer.

A commonly occurring T-to-G polymorphism at nucleotide 309 (T309G) of MDM2 has been the focus of many case-control association studies of ovarian cancer in different ethnic populations. However, these studies indicated different or even conflicting results. Kang et al. found that MDM2 SNP309G allele significantly reduced the risk of ovarian cancer and might be a potential protective factor for ovarian cancer development in Chinese women [32]. But knappskog et al. found that MDM2 SNP309G allele significantly increased the risk of ovarian cancer [23]. So it is worthy to make a meta-analysis to evaluate relationship between MDM2 SNP309 polymorphism and ovarian cancer.

In this meta-analysis, after a critical review of the 6 studies on MDM2 SNP309 polymorphism (a total of 1534 cases and 2211 controls), a comprehensive assessment was performed to investigate whether polymorphisms in MDM2 SNP309 was significantly associated with risk of ovarian cancer. Although no associations between the MDM2 SNP309 polymorphism and ovarian cancer were observed based on total population, significant associations were found in Asian population in subgroup analysis by ethnicity.

The prevalence of homozygous SNP309 variant genotype in Caucasian patients with ovarian cancer was 7.8–17.2% [24], [25], [30], while in healthy Caucasians, the prevalence was 12% [7]. No observable link was established between MDM2 SNP309 and ovarian cancer susceptibility of Caucasian women in two case-control studies [30], [34]. In contrast, the prevalence of the G/G genotype was 31% in healthy Chinese women, and the presence of at least one G-allele significantly decreased the risk for ovarian cancer in Chinese women [32]. In our meta-analysis, the frequency of variant allele MDM2 309G was 46.7%–48.8% among Asian population, and 25.0%–48.8% among Caucasians. This might lead to MDM2 SNP309 polymorphism genotype distribution disequilibrium when all ethnic populations were pooled together. As ethnicity was significantly associated with risk of ovarian cancer, it was essential to conduct a subgroup analysis based on ethnicities.

In the subgroup meta-analysis based on ethnicity, compared with T allele, a significantly reduced risk of ovarian cancer is associated with G allele; compared with TT genotype, a significantly reduced risk of ovarian cancer is associated with GG genotype, TG genotype and the combined TG/GG genotypes in Asian subgroup. Further investigations on large scale on Asian populations may confirm this result. In Caucasian subgroup, no significant association was found in different genetic models. Our results indicate that ethnicity may be a critical factor on the effects of the polymorphic alleles.

Although the case and control groups of the included studies were well controlled by age distribution, smoking habits and menstrual status, there are still a number of limitations in this meta-analysis. First, the analysis did not consider gene-gene and gene-environment interactions because of the lack of sufficient data; second, specific environmental and lifestyle factors may influence the results of this analysis; third, while no publication bias was identified, there is still a possibility that our meta-analysis was biased toward a positive result since negative results were likely to be unreported. In order to provide a more precise estimation by adjustment for confounders, future studies must be taken in larger samples and take potential confounders such as P53 and BRCA1/2 into account.

In summary, positive results have been shown on the search for polymorphic variants influencing the risk of ovarian cancer. This meta-analysis provides evidence of the association between MDM2 309 polymorphism and ovarian cancer risk, supporting the hypothesis that MDM2 SNP309 G allele probably acts as an important ovarian cancer protective factor in Asians, but not in Caucasians. Since the results of our meta-analysis are preliminary and may be biased by the relatively small number of subjects, it still needs to be validated by well-designed studies using larger samples in the future.

## Supporting Information

Checklist S1 (DOC)

Diagram S1 (DOC)
