# Supplementary material for: The MDM2 309T>G Polymorphism and Ovarian Cancer Risk: A Meta-Analysis of 1534 Cases and 2211 Controls
Source: PLoS One. 2013 Jan 31;8(1):e55019. doi: 10.1371/journal.pone.0055019 (PMC3561416; doi:10.1371/journal.pone.0055019)
Supplement: Diagram S1 — (DOC) [file pone.0055019.s002.doc]

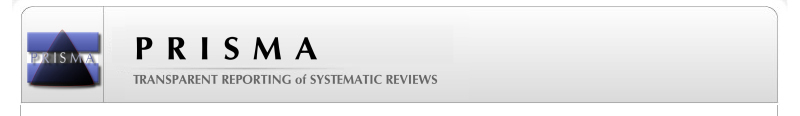
**PRISMA 2009 Flow Diagram**

**Screening**

**Included**

**Eligibility**

**Identification**

Records identified through database searching
(n =15 )

Additional records identified through other sources
(n = 2 )

Records after duplicates removed
(n =16 )

Records screened
(n =16)

Records excluded
(n = 5)

Full-text articles assessed for eligibility
(n = 11)

Full-text articles excluded, with reasons
(n = 3)

Studies included in qualitative synthesis
(n = 8)

Studies included in quantitative synthesis (meta-analysis)
(n = 6)
